# Revolutionizing Medical Microbiology: How Molecular and Genomic Approaches Are Changing Diagnostic Techniques

**DOI:** 10.7759/cureus.47106

**Published:** 2023-10-16

**Authors:** Poyasha A Goyal, Nandkishor J Bankar, Vaishnavi H Mishra, Sonali K Borkar, Jagadish G Makade

**Affiliations:** 1 Microbiology, Datta Meghe Medical College, Datta Meghe Institute of Higher Education and Research (DU), Wardha, IND; 2 Microbiology, Jawaharlal Nehru Medical College, Datta Meghe Institute of Higher Education and Research (DU), Wardha, IND; 3 Community Medicine, Datta Meghe Medical College, Datta Meghe Institute of Higher Education and Research (DU), Wardha, IND; 4 Community Medicine, Datta Meghe Medical College, Datta Meghe Institute of Medical Sciences(DU), Wardha, IND

**Keywords:** revolutionizing healthcare, diagnostic tests, traditional molecular techniques, rt-pcr, molecular diagnostics

## Abstract

Molecular and genomic approaches have revolutionized medical microbiology by offering faster and more accurate diagnostic techniques for infectious diseases. Traditional methods, which include culturing microbes and biochemical testing, are time-consuming and may not detect antibiotic-resistant strains. In contrast, molecular and genomic methods, including polymerase chain reaction (PCR)-based techniques and whole-genome sequencing, provide rapid and precise detection of pathogens, early-stage diseases, and antibiotic-resistant strains. These approaches have advantages such as high sensitivity and specificity, the potential for targeted therapies, and personalized medicine. However, implementing molecular and genomic techniques faces challenges related to cost, equipment, expertise, and data analysis. Ethical and legal considerations regarding patient privacy and genetic data usage also arise. Nonetheless, the future of medical microbiology lies in the widespread adoption of molecular and genomic approaches, which can lead to improved patient outcomes and the identification of antibiotic-resistant strains. Continued advancements, education, and exploration of ethical implications are necessary to fully harness the potential of molecular and genomic techniques in medical microbiology.

## Introduction and background

The healthcare system depends heavily on the science of diagnostics because it serves as the basis for medical judgment. Diagnostic tests provide crucial data that inform personalized cancer treatments, assist in choosing the best antibiotics to treat infections, and offer insightful information on every aspect of healthcare, including disease management, prevention, detection, diagnosis, and treatment [1]. Clinical chemistry, immunology, hematology, microbiology, and molecular diagnostics are some of the primary diagnostic categories. Among these, molecular diagnostics has attracted a lot of attention recently because of its capacity to offer comprehensive insights into diagnosis as well as therapy modalities. This field has experienced significant changes, revolutionizing healthcare by deeply understanding various illness states [2]. Advanced technologies are used in molecular diagnostics to find and examine genetic and molecular changes linked to diseases. To better understand and treat a variety of medical diseases, molecular diagnostics has created new pathways for study and treatment by analyzing people's genetic makeup [3]. Specific diseases and disorders have been addressed for in-depth examination and therapy in the field of molecular diagnostics. This method has made it possible to create precise and personalized therapies based on a person's genetic profile, increasing the effectiveness and results of treatments. Particularly helpful applications of molecular diagnostics include oncology, infectious diseases, genetic abnormalities, and personalized treatment [4]. Two novel therapeutic modalities, pharmacogenomics, and nutrigenomics, have also been made possible by the science of molecular diagnostics. Pharmacogenomics entails examining a person's genetic variants to ascertain how they will react to specific pharmaceuticals, optimizing treatment regimens, and reducing negative drug reactions. Nutrigenomics, on the other hand, investigates how a person's genes and diet interact, enabling tailored nutritional recommendations that can have an impact on disease management techniques [5,6]. Diagnostics and healthcare have undergone a paradigm shift due to molecular diagnostics. Utilizing cutting-edge technologies and expanding our knowledge of genetic and molecular variables has changed medical research, diagnosis, and treatment methods. The potential of molecular diagnostics in creating tailored and successful disease treatment strategies is further highlighted by the development of nutrigenomics and pharmacogenomics [7,8]. The objective of this review is to explore molecular and genomic techniques' potential for improving medical microbiology.

## Review

Search strategy

We used the PubMed and Scopus advanced search strategy to obtain articles from PubMed and Scopus employing the following terms: (“molecular diagnostic techniques” OR “genomic diagnostic techniques” OR “traditional diagnostic techniques" or "molecular and genomic approaches” OR “traditional diagnostic techniques” OR “molecular and genomic approaches” OR “advantages of molecular and genomic approaches in medical microbiology” OR “strengths molecular and genomic approaches” OR “limitations molecular and genomic approaches”). We obtained the most pertinent research papers and used them in different arrangements using the Boolean operators “AND” and “OR.” 

Search outcomes

**Figure 1:**
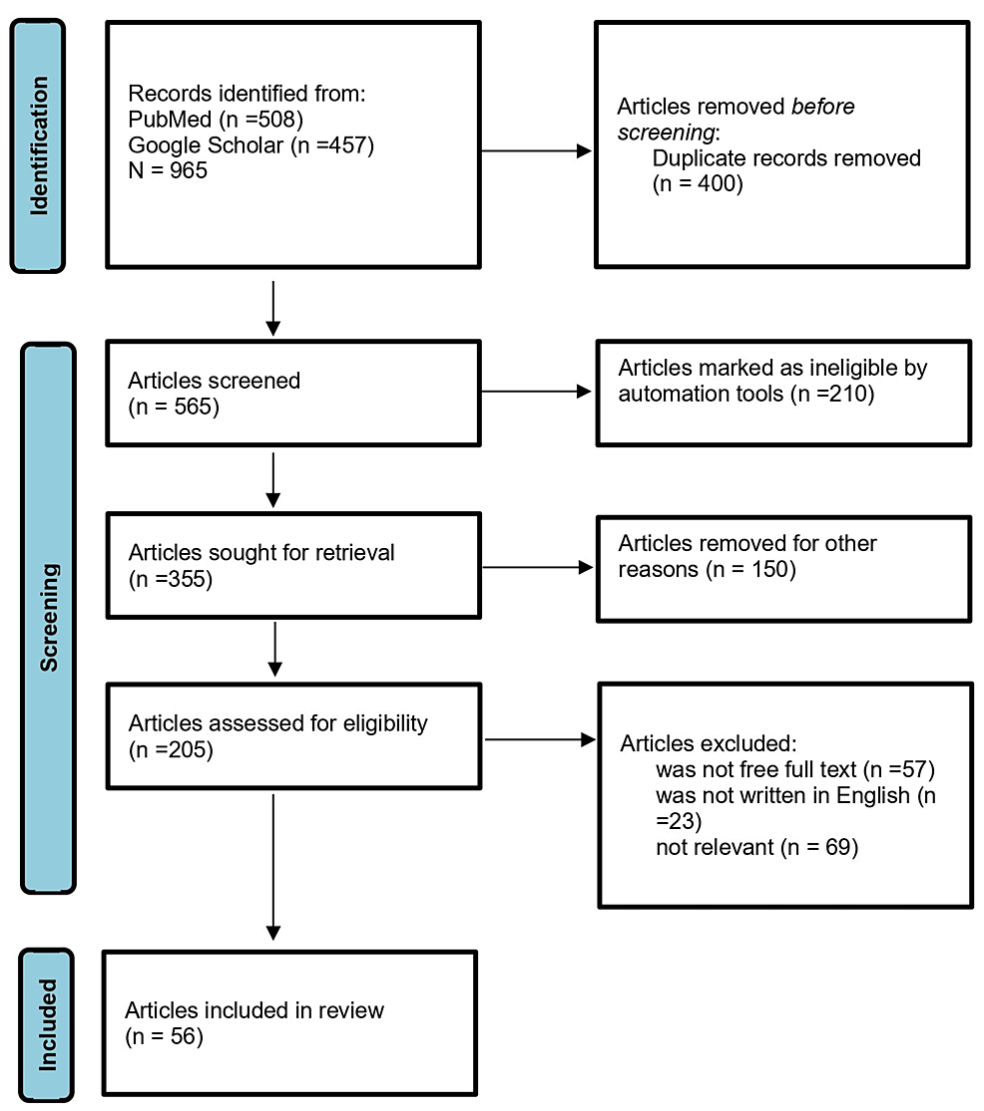
A PRISMA flow chart of the study selection process PRISMA: Preferred Reporting Items for Systematic Reviews and Meta-Analyses; n: number of studies

After conducting the initial search, we identified a total of 965 articles across the searched databases. We then excluded duplicates (n=400) and conducted an initial screening of titles and abstracts, which excluded a further 565 articles. After the full-text screening of the remaining 360 articles, we excluded 149 articles for not meeting the inclusion criteria; the full free text was not available and was not written in English, leaving a total of 56 articles for the final review (Figure 1).

History

The timeline illustrates key milestones in the field of molecular diagnostics. Pauling's term "molecular disease" in 1949 linked genetics to sickle cell anemia. The 1980s saw prenatal tests using deoxyribonucleic acid-cutting enzymes. Companies like Molecular Diagnostics Inc. emerged, followed by gene discoveries and sequencing techniques in the 1990s. The Association for Molecular Pathology (AMP) was formed in 1995; the European Union (EU) allowed gene patents in 1998; and journals like The Journal of Medical Diagnostics started. Legal battles over gene patents occurred, including the 2013 United States Supreme Court ruling against patenting natural gene sequences. The history of molecular and genomic technique advancements is mentioned in Table 1.

**Table 1:** Timeline representing the development of molecular diagnostic techniques DNA: deoxyribonucleic acid; AMP: Association for Molecular Pathology; BRCA1: BReast CAncer gene 1; BRCA2: BReast CAncer gene 2; RNA: ribonucleic acid; CRISPR: clustered regularly interspaced short palindromic repeats [9-11]

Year	Milestone
1949	Introduction of the term "molecular disease" by Pauling et al., linking a single amino acid change to sickle cell anemia
1980	Recommendation of a prenatal genetic test for thalassemia based on restriction enzymes cutting DNA at specific sequences
1980’s	The emergence of companies like Molecular Diagnostics Incorporated and Bethesda Research Laboratories Molecular Diagnostics
1990’s	Identification of newly discovered genes and development of new techniques for DNA sequencing
1995	Formation of the Association for AMP
1998	The European Union's Directive 98/44/EC allows patents on DNA sequences.
1999	Co-founding of The Journal of Medical Diagnostics by AMP
2001	Launch of Expert Reviews in Medical Diagnostics by Informa Healthcare
2002	Commencement of the Hap Map Project to study genetic differences in the human population
2010	The lawsuit by AMP against Myriad Genetics over gene patents related to BRCA1 and BRCA2
2012	Molecular diagnostic techniques for thalassemia utilize genetic hybridization tests.
2013	The US Supreme Court ruled that naturally occurring gene sequences cannot be patented.
2015	CRISPR-Cas9 gene editing, precision medicine initiatives, and liquid biopsies for cancer detection
2018	Single-cell RNA sequencing and liquid biopsies for cancer detection
2020	Genomic epidemiology tracks and contains outbreaks.
2022	Functional genomics identifies therapeutic targets.

Traditional diagnostic techniques versus molecular and genomic approaches

Traditional diagnostic techniques for infectious diseases include culturing microbes on agar plates, biochemical testing, and microscopic examination of samples. These methods are slow, often taking days or weeks to yield results, and they may not be able to identify the causative agent of infection [12]. Additionally, traditional diagnostic techniques may be unable to detect antibiotic-resistant strains of bacteria, which can lead to inappropriate treatment and potentially fatal outcomes [13,14]. Traditional methods' primary drawback is how time-consuming they are. To allow for the growth and identification of organisms, an incubation duration of several days or even weeks is necessary when cultivating bacteria on agar plates. This wait for findings can delay the start of therapy, which can be harmful, especially in cases of serious or life-threatening infections [15].

In contrast, molecular and genomic approaches offer faster and more accurate results and can detect antibiotic-resistant strains of bacteria [16]. These methods entail isolating nucleic acids from a patient's sample, such as blood or tissue, and using molecular and genomic instruments to pinpoint the infection's causal culprit. These techniques include bioinformatics, whole-genome sequencing (WGS), and polymerase chain reaction (PCR) [17]. The DNA testing was significantly accelerated and made more efficient thanks to next-generation sequencing (NGS), which offered a strong alternative for identifying mutations [18].

Advantages of molecular and genomic approaches

In comparison to conventional diagnostic methods, molecular and genomic approaches have several advantages (Table 2).

**Table 2:** The strengths and limitations of traditional diagnostic techniques and molecular/genomic approaches in medical diagnostics [2]

Diagnostic technique	Strengths	Limitations
Traditional diagnostic techniques	Familiar and widely used	Limited sensitivity and specificity
	Non-invasive and low-cost	Longer turnaround time
	Accessible in resource-limited settings	May not detect early-stage diseases
	Provides visual information through imaging and microscopy	Limited ability to identify antibiotic-resistant strains
Molecular and genomic approaches	High sensitivity and specificity	Requires specialized equipment and expertise
	Rapid detection and diagnosis	Higher cost compared to traditional techniques
	Ability to detect early-stage diseases	Data interpretation and analysis can be complex
	Potential for targeted therapies and personalized medicine	Standardization challenges across different platforms/methods
	Identification of antibiotic-resistant strains	Ethical considerations related to genetic information

These methods are quicker and more precise. While whole-genome sequencing can offer a thorough examination of all the bacteria present in a sample, PCR-based approaches can pinpoint the infection's primary cause in a matter of hours. Early and more focused treatment of infections is made possible by this speed and accuracy, which can improve patient outcomes [19,20]. Traditional diagnostic methods are less sensitive and specific than molecular and genomic approaches. These techniques can identify microorganisms at low concentrations that conventional techniques can miss [21]. Furthermore, molecular and genomic techniques can be used to identify particular strains of bacteria, viruses, and fungi, which can be very helpful in infectious disease epidemics [22].

Antibiotic-resistant bacterial strains can be found using molecular and genomic methods. These strains are spreading more widely, and conventional diagnostic methods might not be able to distinguish them. Antibiotic-resistant genes can be found using molecular and genomic methods, allowing for effective infection therapy [23,24].

Molecular and genomic techniques in the diagnosis of infectious diseases

Molecular and genomic techniques are being used in the diagnosis of a wide range of infectious diseases [25]. Polymerase chain reaction-based methods are commonly used to diagnose viral infections, such as human immunodeficiency virus (HIV), hepatitis, and influenza [26]. These methods can also be used to diagnose bacterial infections, such as tuberculosis, chlamydia, and gonorrhea [27]. Another method that is rapidly being employed in medical microbiology is whole-genome sequencing. This technique includes sequencing every piece of DNA found in a sample of microorganisms, which can produce a thorough examination of every bacterium present [28]. When infectious disease outbreaks occur, whole-genome sequencing can be very helpful in locating the outbreak origin and monitoring the disease progress [29].

Nucleic acid amplification test 

The gold standard for amplification procedures in diagnostics is PCR. Since the method's initial publication in 1985 [30]. In medical microbiology, PCR-based methods are frequently employed to identify bacterial and viral illnesses. These procedures entail amplifying particular DNA sequences from a patient's sample, which can then be found using a variety of techniques, including gel electrophoresis or fluorescence. Gene analysis, the diagnosis of numerous genetic illnesses, and the detection of bacterial, viral, and fungal infections have all greatly benefited from the invention of methods for amplifying DNA segments [31-34]. Polymerase chain reaction-based methods are quick and accurate, and they can find small amounts of bacteria [35]. The detection of bacterial strains resistant to antibiotics is also possible using PCR-based methods. These strains frequently possess particular genes that confer antibiotic resistance, and PCR-based methods can identify the presence of these genes. This can aid in determining the most effective way to treat infections [36].

Whole-genome sequencing

A potent technique for the diagnosis of infectious disorders is WGS. This technique entails sequencing every piece of DNA found in a sample of microorganisms, which can produce a thorough examination of every bacterium present. It allows for the high-resolution characterization of bacterial pathogens in terms of traits including pathogenicity, molecular epidemiology, and antibiotic resistance [37]. When infectious disease outbreaks occur, whole-genome sequencing can be very helpful in locating the outbreak's origin and monitoring the disease's progress [38,39]. The identification of bacterial strains resistant to antibiotics is another application of WGS. This method involves identifying specific genes that confer antibiotic resistance, which can aid in determining the most effective way to treat infections [40]. The development of next-generation sequencing equipment has decreased the cost of WGS. However, the absence of a WGS beginner's protocol still prevents its acceptance in some contexts [37].

In bioinformatics and data analysis, molecular and genomic approaches and numerous data sets are produced by molecular and genomic techniques, and these data sets need to be evaluated and understood. Medical microbiology uses molecular and genomic techniques, while bioinformatics is the branch of science that deals with the processing and interpretation of biological data [41,42]. In bioinformatics, biological data are analyzed and interpreted using computational tools and algorithms. The identification of certain genes and proteins, sequence comparison, and biological molecule structure and function predictions are all possible with the use of these technologies. For the processing and interpretation of the enormous volumes of data produced by molecular and genomic techniques, bioinformatics is crucial [43].

Matrix-assisted laser desorption ionization time-of-flight mass spectrometry

The 1980s saw the development of matrix-assisted laser desorption ionization time-of-flight mass spectrometry (MALDI-TOF) by Hillenkamp and Karas, which is essential for analyzing biomolecule masses. It ionizes molecules using a matrix and laser to create distinctive profiles for proteomics, biomolecule analysis, and microbiological identification. Their laser-ionization and time-of-flight detection methods are used in clinical diagnostics, drug development, and quality control because they provide quick, precise molecular insights. It quickly rose to prominence in clinical microbiology, revolutionizing the identification of microorganisms based on mass spectra, "fingerprints," and proteomics, detecting and characterizing proteins [44]. It evolved, advancing sample processing, instrument design, and data analysis for use in a variety of industries, including environmental analysis, food safety, and drug discovery. Due to its great sensitivity, speed, and versatility, MALDI-TOF has become a cornerstone of mass spectrometry and is essential to modern research across many fields of science [45].

Next-generation sequencing

The field of genetic research has been completely changed by NGS, also known as massively parallel or deep sequencing. This technology makes it possible to quickly and affordably sequence complete genomes, opening the door for ground-breaking research in a variety of domains. Next-generation sequencing has ushered in a new era of genomics research by making it possible to sequence the human genome in just one day, enabling improvements in personalized medicine, evolutionary biology, microbiology, and other fields. This review examines the foundations, uses, and consequences of NGS, emphasizing its contribution to advancing knowledge and its potential to alter how we perceive the genetic universe [46]. Next-generation sequencing has revolutionized genomics. After a modest start in 1977 with Sanger sequencing, NGS developed in the 2000s, characterized by 454's pyro-sequencing and Illumina's parallel technology [47]. Ion Torrent's semiconductor detection, Pac Bio's real-time readings, and Oxford Nanopore's nanopore-based sequencing have all contributed to the advancement of NGS. This revolution affected genetics, medicine, agriculture, and other fields through initiatives like the Human Genome Project. Genomic profiling, cancer detection, infectious disease detection, transcriptome analysis, epigenetic, non-invasive prenatal testing (NIPT), pharmacogenomics, diagnosis of rare diseases, microbiome research, and human leukocyte antigen (HLA) typing are a few of the uses of NGS. Next-generation sequencing has issues with data handling yet continues to advance molecular diagnostics and tailored treatment while providing speed, accuracy, and scalability.

Challenges in implementing molecular and genomic approaches

Although molecular and genomic approaches have several benefits over conventional diagnostic methods, there are also several practical obstacles [48]. Some medical facilities might not have the necessary tools and knowledge that these methods demand. Additionally, these methods might be pricey, which might restrict who can use them [49]. Numerous data sets are produced by molecular and genomic techniques, and these data sets need to be evaluated and understood. This calls for specific knowledge in data processing and bioinformatics, which might not be available in all medical facilities [50]. The use of molecular and genomic methods in medical microbiology has ethical and legal implications. Concerns including patient privacy, informed consent, and the ownership and usage of genetic data are among them [51].

Future of molecular and genomic approaches

Molecular and genomic methods are anticipated to influence the direction of medical microbiology in the future. With these methods, infectious illness diagnoses can be made more quickly and precisely, which can lead to better patient outcomes [52,53]. Additionally, in light of the increasing prevalence of antibiotic resistance, molecular and genomic techniques can be used to discover bacterial strains that are resistant to antibiotics [54].

Future developments are projected to increase the accessibility and availability of molecular and genomic methods. It will be necessary to do this by creating new technology and educating medical personnel on how to use it [55]. Additionally, continued investigation into the ethical and legal issues connected to the application of molecular and genomic techniques in medical microbiology will be necessary [56].

## Conclusions

Medical microbiology is undergoing a revolution thanks to molecular and genomic techniques. Infectious disease diagnoses can now be made more quickly and precisely thanks to these methods, which can benefit patient outcomes. Additionally, molecular and genomic techniques can aid in the identification of bacterial strains that are resistant to antibiotics, which is becoming more crucial in light of the rise in antibiotic resistance. Molecular and genomic techniques are likely to be made more broadly available and accessible in the future, notwithstanding the difficulties involved in their implementation. This will necessitate continual study and development as well as instruction in the usage of these methods for medical practitioners. In the end, the application of molecular and genomic methods in medical microbiology can revolutionize how we identify and treat infectious diseases while also enhancing patient outcomes globally.
